# Estimation and Prognostic Role of Prostate-specific Antigen (PSA) Doubling Time After Radical Prostatectomy

**DOI:** 10.1016/j.euros.2026.05.006

**Published:** 2026-05-21

**Authors:** Pietro Scilipoti, Max Callenmark, Hans Garmo, Pär Stattin, Rolf Gedeborg, Marcus Westerberg

**Affiliations:** a Department of Experimental Oncology/Unit of Urology, URI, IRCCS Ospedale San Raffaele, Milan, Italy; b Vita-Salute San Raffaele University, Milan, Italy; c Department of Surgical Sciences, Uppsala University, Uppsala, Sweden

**Keywords:** Doubling time, Detection limit, Prostate-specific antigen, Prostate cancer, Radical prostatectomy

## Abstract

**Background and objective:**

There are several methods for estimating prostate-specific antigen (PSA) doubling time (DT), and many men exhibit long periods of undetectable PSA below 0.1 ng/ml after radical prostatectomy (RP). We quantified between-method differences in estimated PSA-DT at PSA relapse after RP and evaluated how these differences affect discrimination of prostate cancer (PCa) death.

**Methods:**

Men who underwent RP in 2007–2024 and subsequently developed a PSA relapse, defined as two consecutive values above 0.2 ng/ml, were included. We estimated PSA-DT from the date of the first undetectable PSA after RP until PSA relapse using four methods: (1) all detectable PSAs only, (2) two most recent detectable PSAs, (3) three most recent PSAs (with the third forced to 0.1 ng/ml if ≤0.1 ng/ml), and (4) all PSAs using a missing data approach. Discrimination of 10-yr PCa death was assessed using concordance index (C-index).

**Key findings and limitations:**

A total of 3826 men were included. Median PSA-DT ranged from 4.4 to 8.6 mo, with a wide range in PSA-DT across different estimation methods. All four methods discriminated PCa death to a similar level, with C-index ranging from 0.70 to 0.74, which was slightly lower than the C-index (0.77) for the absolute PSA level at relapse. This study was restricted to men with PSA relapse after RP and may not be generalizable to other patient categories.

**Conclusions and clinical implications:**

PSA-DT after RP was sensitive to the choice of estimation method, and all methods had comparable prognostic value to the last measured PSA level.

## Take Home Message

Prostate-specific antigen doubling time at relapse after radical prostatectomy is a predictive of outcome; however, estimates of doubling time varied substantially depending on the estimation method. Discriminative performance was similar across methods and was comparable to PSA alone at relapse.

ADVANCING PRACTICE **What does this study add?**

This study evaluated different methods for estimating prostate-specific antigen (PSA) doubling time (PSA-DT) after PSA relapse following radical prostatectomy. The four estimation approaches yielded substantially different PSA-DT values at the individual level. Despite this variability, all methods showed similar ability to discriminate prostate cancer death, comparable to that of the absolute PSA level at relapse. These findings highlight inherent methodological challenges in calculating PSA-DT after radical prostatectomy and suggest that the absolute PSA level at relapse provides a comparable prognostic discrimination.

**Clinical Relevance**

PSA doubling time (PSA-DT) is increasingly used to guide risk stratification and treatment decisions after radical prostatectomy, making accurate measurement and interpretation clinically important. This study demonstrates that PSA-DT estimates vary substantially depending on the calculation method used, despite similar prognostic performance across methods. These findings highlight the need for greater standardization and awareness regarding PSA-DT assessment when using PSA kinetics to support clinical decision-making in prostate cancer management. Associate Editor: Roderick C.N. van den Bergh.

**Patient Summary**

When PSA level starts to rise after RP, PSA-DT describes how quickly the PSA level is increasing and may predict the risk of PCa death. In this study, the PSA-DT changed a lot depending on how it was calculated and had comparable prognostic value to the last measured PSA level.

## 1 Introduction

After radical prostatectomy (RP) for prostate cancer (PCa), serum levels of prostate-specific antigen (PSA) should become undetectable. However, one out of three men will experience a PSA relapse, ie, PSA level becomes detectable or starts to increase [1]. PSA doubling time (PSA-DT) has a key prognostic role in men with a PSA relapse after RP and is used for risk stratification, according to the European Association of Urology (EAU) guidelines [2], [3], [4], [5].

A plethora of methods have been used to calculate PSA-DT in different settings and they have led to different estimates of PSA-DT [6], [7]. The estimation of PSA-DT in men with a PSA relapse after RP is particularly intricate since many men have several PSA values below the detection limit (typically 0.1 ng/ml) before PSA becomes detectable. Some men may also have a negative PSA-DT (eg, PSA level decreases).

It is unknown to what extent the previously observed range of PSA-DT estimates across different estimation methods applies to men with a PSA relapse after RP. The estimates may also differ in their ability to predict PCa death based on how many values that are used in the computation, how values below the detection limit are handled, and how negative estimates are handled. It is also not clear how the method’s discriminatory ability compares to that of other prognostic indicators, such as absolute PSA level at relapse. Systematic bias of the estimation methods when PSA follows an exponential growth curve in this context is also unknown.

We aimed to quantify between-method differences in estimated PSA-DT at PSA relapse after RP, arising from the use of different estimation approaches, and to evaluate how these differences affect discrimination of PCa death when compared with PSA level at relapse, pathological Gleason score, and time to relapse. We additionally assessed the impact of estimation methods on group-level estimates of PCa death within predefined PSA-DT categories and evaluated method-specific estimation bias using simulation studies.

## 2 Materials and methods

### 2.1 Data sources

In Prostate Cancer data Base Sweden (PCBase), the National Prostate Cancer Register (NPCR) of Sweden has been linked to other health care registers and demographic databases, including the Patient Register, the Prescribed Drug Register, the Total Population Register, and the Cause of Death Register. In PCBase Xtend (extended treatments and endpoints), longitudinal data on PSA and use of androgen-deprivation therapy (ADT; any use of gonadotropin-releasing hormone agonist or first-generation anti-androgen [bicalutamide]) has been extracted from the health care IT systems [8].

### 2.2 Variables

We extracted Gleason score at RP, T and N stage at RP, use of postoperative radiotherapy, PSA at date of diagnosis and during follow-up, and use of ADT during follow-up. Relapse was only possible if a man had a PSA response, defined as at least one PSA value ≤0.1 ng/ml within 42 d after RP, or if the first PSA after RP was measured after 42 d and was ≤0.1 ng/ml. Date and cause of death (PCa and other causes) were extracted from the Cause of Death Register.

### 2.3 Definitions of PSA relapse

We considered two consecutive PSA values above the detection limit of 0.1 ng/ml, in line with the national comprehensive cancer network and the Swedish guidelines for PCa [9], [10], and 0.2 ng/ml [2] (Supplementary Fig. 1). Men who did not have an undetectable PSA level after RP or who initiated ADT or salvage radiotherapy (RT) before a documented PSA relapse, were not considered as having a PSA relapse [1].

In complementary analyses, we required only one value above 0.1/0.2 ng/ml to define relapse.

### 2.4 Study population

Men diagnosed with PCa and registered in NPCR, who underwent RP in the period 2007–2024, who resided in a region in Sweden where longitudinal data on PSA and ADT was available at the time of RP, and who had a PSA relapse were included in the analyses. Follow-up for death after relapse started on the date of relapse and ended on the date of death, on migration, or on December 31, 2024.

### 2.5 Estimation of PSA-DT

PSA-DT was estimated using four different methods. They were all based on PSA values measured after the first PSA ≤0.1 ng/ml following RP, until and including the PSA value defining relapse:

**Detectable PSAs**: all detectable PSAs above 0.1 ng/ml were used in the estimation.
**Two most recent PSAs**: the two most recent PSAs were used (including PSA at relapse).
**Three most recent PSAs**: the three most recent PSAs were used (including PSA at relapse), where in case the second-to-last PSA was ≤0.1 ng/ml, it was set to 0.1 ng/ml, despite the true value being unknown, as described in [1].
**All PSAs with censored maximum likelihood estimation (MLE)**: all PSA values were used, and undetectable PSA values (≤0.1 ng/ml) were handled as censored data (ie, as ≤0.1 ng/ml) in a MLE approach to the missing data.

PSA-DT was estimated separately for each man using log(2)/slope of a linear regression model fitted to the dates of the tests and the logarithm of the PSA values, using each of the four methods (see Supplementary Materials for details). Note that the estimation methods may occasionally produce negative PSA-DTs.

Estimated PSA-DTs above 100 mo were set to 100 mo. A negative PSA-DT indicates a decreasing PSA level, which we a priori expected to be similar to very long PSA-DTs rather than short PSA-DTs. To illustrate the proportion and impact of negative PSA-DTs, we set PSA-DTs below –100 mo to –100 mo.

### 2.6 Statistical analysis

We compared the estimated PSA-DTs on an individual level in scatter plots, and assessed discrimination of PCa-specific death within 10 yr from relapse (by censoring for death from other causes), using the concordance index (C-index) with a 95% confidence interval (CI). A negative PSA-DT indicates a decreasing PSA level that we a priori expected to be comparable to very long PSA-DTs; thus, we additionally performed analyses of discrimination of PCa death where negative PSA-DTs were set to 100 mo. We also assessed discrimination of PSA at relapse, time to relapse, and pathological Gleason score. These analyses were performed overall and in men with high-risk PCa at RP (Gleason 8–10, PSA >20 ng/ml, T3–4, or N+).

To assess group-level estimates of the risk of PCa death, we estimated the 10-yr risk of death from PCa and from other causes using competing risk cumulative incidence curves with a 95% CI, according to PSA-DT (below <0, 0–6, 6–12, 12–24, and above 24 mo).

In simulations studies, simulated PSA trajectories were generated according to an exponential growth curve for 100 000 men based on a constant (intercept), time, and PSA-DT. We evaluated the bias of each estimator of PSA-DT according to various intercepts, PSA-DTs, and standard deviations of the normal distribution. For details, see Supplementary Materials.

## 3 Results

### 3.1 Inclusion, baseline characteristics, and follow-up

There were 7691 men with a PSA relapse after RP based on two values above the cutoff of 0.1 ng/ml (Supplementary Fig. 2). The majority of men (84%) had a Gleason score of 6–7 in the assessment of the postoperative specimen. Median time to relapse was 31 mo (interquartile range [IQR]: 16–54 mo), and median number of PSA tests below 0.1 ng/ml was 5 (IQR: 3–8) (Table 1). Median time between PSA measurements was 156 d (IQR: 91–203 d), and 60% of measurements were taken within 180 d of the previous measurement and 92% within 1 yr.

**Table 1:** Baseline characteristics in men with PSA relapse according to two definitions of relapse

	Two consecutive values above 0.1 ng/ml		Two consecutive values above 0.2 ng/ml	
	*N*	(%)	*N*	(%)
Men (*N*)	6767	(100)	3826	(100)
Age at RP				
Median (IQR)	66 (62–70)		67 (62–70)	
Pathological Gleason score				
6	673	(10)	381	(10)
7 (3+4)	2835	(42)	1524	(40)
7 (4+3)	2168	(32)	1266	(33)
8	570	(8)	366	(10)
9–10	521	(8)	289	(8)
PSA before RP (ng/ml)				
Median (IQR)	7.7 (5.4–12.0)		7.8 (5.4–12)	
Time to relapse (mo)				
Median (IQR)	31 (16–54)		38 (20–66)	
Number of PSA tests between first undetectable PSA and relapse				
Median (IQR)	7 (5–10)		9 (6–13)	
Number of PSA tests ≤0.1 ng/ml				
Median (IQR)	5 (3–8)		4 (2–7)	
PSA at relapse (ng/ml)				
Median (IQR)	0.2 (0.1–0.2)		0.3 (0.2–0.4)	
PSA doubling time (mo)–detectable PSAs				
Median (IQR)	5.0 (0.2–12.9)		8.6 (3.8–16.7)	
<0	1688	(25)	252	(7)
0–12	3262	(48)	2178	(57)
>12	1817	(27)	1396	(36)
PSA doubling time (mo)–two most recent PSAs				
Median (IQR)	4.7 (–1.1–11.7)		4.4 (–0.8–11.8)	
<0	1749	(26)	980	(26)
0–12	3359	(50)	1907	(50)
>12	1659	(25)	939	(25)
PSA doubling time (mo)–three most recent PSAs				
Median (IQR)	11.8 (6.4–22.8)		7.1 (4.1–11.7)	
<0	3	(0)	3	(0)
0–12	3418	(51)	2908	(76)
>12	3346	(49)	915	(24)
PSA doubling time (mo)–all PSAs with censored MLE				
Median (IQR)	8.1 (4.1–16.6)		7.9 (4–14.1)	
<0	1	(0)	0	(0)
0–12	4389	(65)	2630	(69)
>12	2377	(35)	1196	(31)

IQR = interquartile range; MLE = maximum likelihood estimation; PSA = prostate-specific antigen; RP = radical prostatectomy.

Median follow-up for death after relapse was 6.0 yr (IQR: 3.8–8.8 yr) (Supplementary Table 1).

Baseline characteristics and follow-up in men with a PSA relapse defined by the cutoff of 0.2 ng/ml (*n* = 3826) were comparable to those obtained using a cutoff of 0.1 ng/ml (Table 1, Supplementary Table 1). More men were excluded, as they started ADT or salvage RT before a documented relapse, when 0.2 ng/ml was used as cutoff (*N* = 2775 men), compared to those using 0.1 ng/ml as cutoff (*N* = 940 men) (Supplementary Fig. 2).

### 3.2 Individual-level range of PSA-DT estimates across estimation methods

**Fig. 1:**
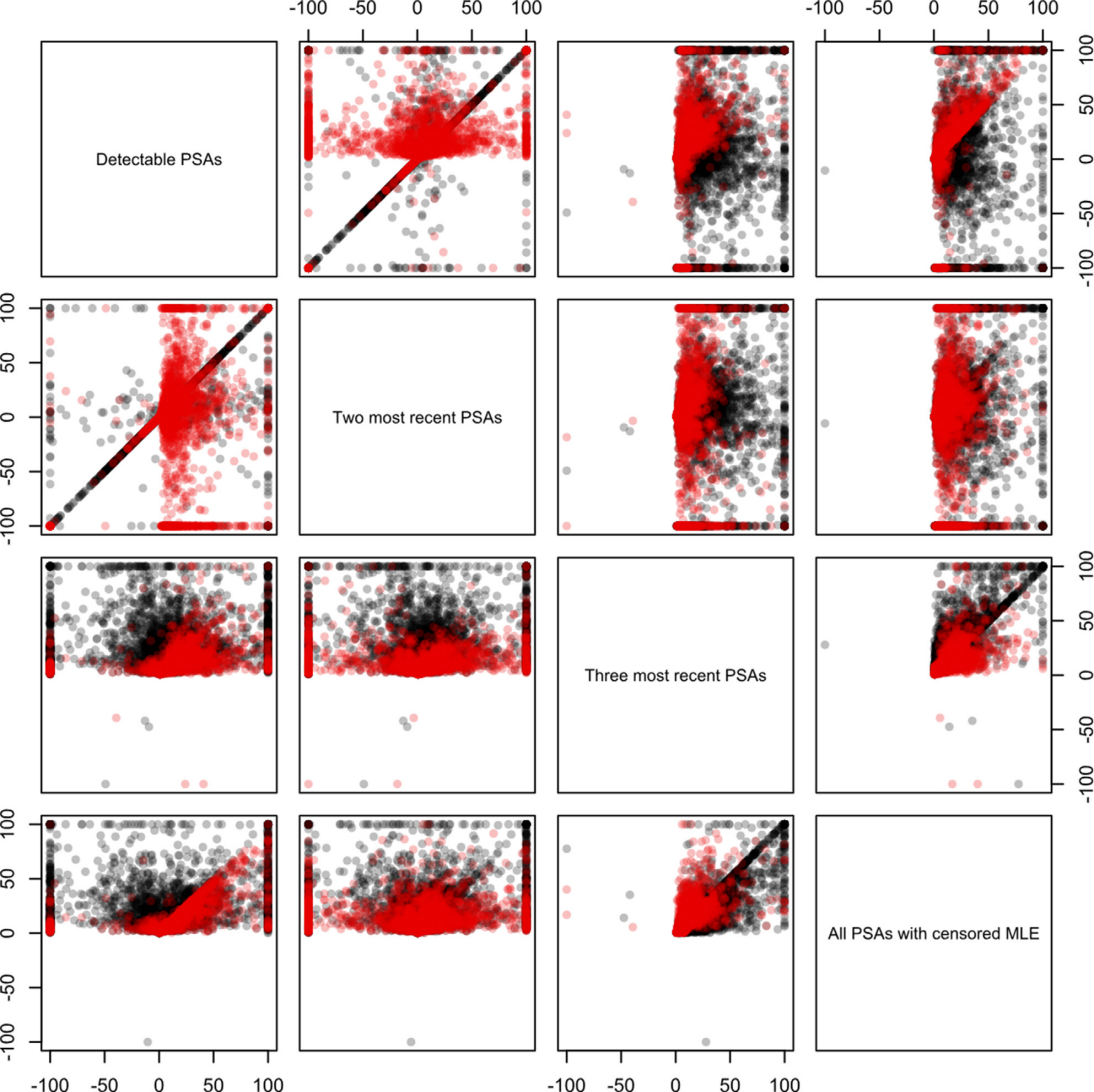
Scatter plot comparing estimates of PSA doubling time at PSA relapse, according to two definitions of relapse. MLE = maximum likelihood estimation; PSA = prostate-specific antigen.

There was a wide individual-level range in the estimated PSA-DTs across the four estimators (Fig. 1). When using 0.1 ng/ml as cutoff, the median PSA-DT time ranged from 4.7 mo, using the two most recent PSAs, to 11.8 mo, using the three most recent PSAs, and when using 0.2 ng/ml as cutoff, the median PSA-DT time ranged from 4.4 to 8.6 mo (Table 1).

### 3.3 Discrimination of risk of PCa death

**Fig. 2:**
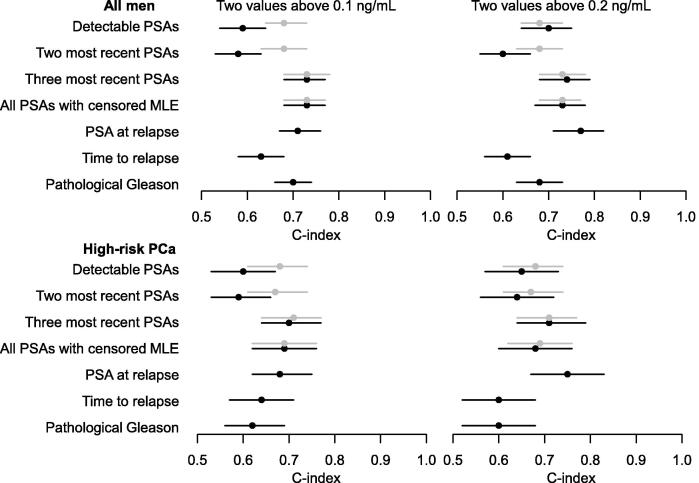
Discrimination of PCa death within 10 yr from PSA relapse, according to two definitions of relapse. Grey indicates that negative PSA doubling times were set to the maximum (100 mo) and black indicates that negative PSA doubling times remained negative. Men were censored if they died from other causes. Gleason score was used as an ordinal variable with values 1: Gleason 6; 2: Gleason 3+4; 3: Gleason 4+3; 4: Gleason 8; and 5: Gleason 9–10. The subgroup analyses of men with high-risk PCa were based on *N* = 1700 men when using a cutoff of 0.1 ng/ml and *N* = 1010 men when using a cutoff of 0.2 ng/ml. C-index = concordance index; MLE = maximum likelihood estimation; PCa = prostate cancer; PSA = prostate-specific antigen.

The discriminatory ability of the estimates of PSA-DTs varied slightly according to method but to a lesser extent when values below 0 mo were set to 100 mo (Fig. 2). When using 0.1 ng/ml as cutoff, discriminatory performance was poor when all detectable PSAs (C-index 0.59, 95% CI: 0.55–0.64) and the two most recent PSAs (C-index 0.58, 95% CI: 0.53–0.63) were included; whereas discrimination was substantially higher when the three most recent PSAs (C-index 0.73, 95% CI: 0.68–0.87) and all PSAs with censored MLE (C-index 0.73, 95% CI: 0.68–0.87) were included. Upon setting the negative PSA-DTs to 100 mo, the discriminatory performance of PSA-DT increased when all detectable PSAs (C-index 0.68, 95% CI: 0.64–0.73) and the two most recent PSAs (C-index 0.68, 95% CI: 0.63–0.72) were included.

Results were similar when using 0.2 ng/ml as cutoff, with C-indices between 0.70 and 0.74 for PSA-DT and 0.77 for the absolute PSA value at relapse (Fig. 2). These patterns were similar in the subgroup of men with high-risk PCa.

### 3.4 Group-level estimates of risk of PCa death according to DT

**Fig. 3:**
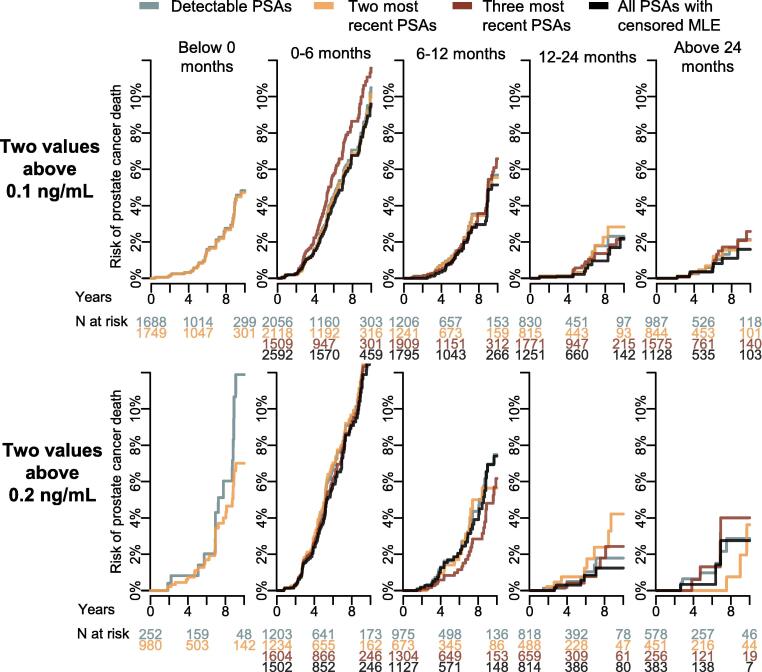
Risk of prostate cancer death according to doubling time at PSA relapse, using two definitions of relapse. Death from other causes was handled as a competing risk. Negative PSA doubling times were assigned to the low-risk relapse group. MLE = maximum likelihood estimation; PSA = prostate-specific antigen.

The 10-yr cumulative incidence of PCa death was 6.1% (95% CI: 5.8–6.5) overall and varied modestly according to the PSA-DT estimation method when using PSA cutoff of 0.1 ng/ml (Fig. 3). Among men classified as having a negative PSA-DT by methods that frequently yielded negative estimates (detectable PSAs and the two most recent PSAs), the 10-yr cumulative incidence ranged from 4.7% to 4.8%, which was slightly lower than that observed in men with a PSA-DT of 6–12 mo, for whom estimates ranged from 5.1% to 6.6%. Results were similar when using a PSA cutoff of 0.2 ng/ml.

### 3.5 Simulation studies of systematic bias

**Fig. 4:**
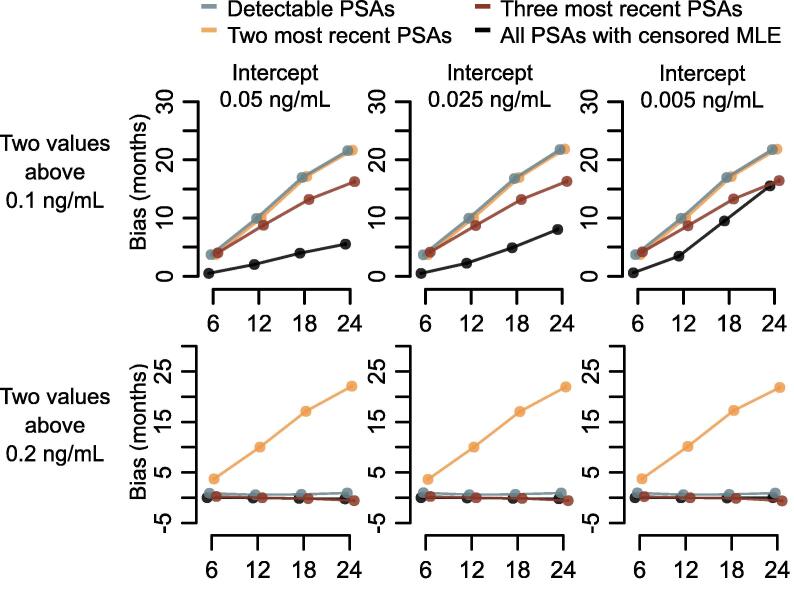
Bias of each estimator in a simulation study where PSA followed an exponential growth curve defined by an initial PSA (intercept) and PSA doubling time, with a standard deviation of 0.1 ng/ml for the normally distributed log-PSA values, according to two definitions of relapse. MLE = maximum likelihood estimation; PSA = prostate-specific antigen.

The simulation studies indicated that all methods tended to overestimate PSA-DT, and this bias was larger for longer PSA-DTs (particularly for 18 and 24 mo) compared to shorter PSA-DTs when using a PSA cutoff of 0.1 ng/ml (Fig. 4). Bias was generally the smallest when using all PSAs with censored MLE to estimate PSA-DT**.** For a PSA-DT of 12 mo, with an intercept of 0.025 ng/ml and a standard deviation of 0.10 ng/ml, the bias ranged from 2.2 mo, when using all PSAs with censored MLE, to 10 months, when using the two most recent PSAs.

The method based on the two most recent PSAs was clearly biased when using a PSA cutoff of 0.2 ng/ml but the other methods were not.

### 3.6 Complementary analyses

Similar findings were reported when relapse was defined using one PSA value above the cutoff (Supplementary Table 2, Supplementary Fig. 3 and 4).

## 4 Discussion

There was a wide individual-level range of PSA-DT estimates across different estimation methods in men with PSA relapse after RP for PCa. Some methods produced negative values in around one quarter of men, and these men had a higher risk of PCa death than men with PSA-DT above 12 mo. The ability of PSA-DT to discriminate risk of PCa death was largely similar across different methods and to PSA at relapse. All methods tended to overestimate PSA-DT in simulations when using a PSA cutoff of 0.1 ng/ml for relapse, particularly when the true PSA-DT was 18 and 24 mo. Only the method based on two most recent PSAs was biased when using the PSA cutoff of 0.2 ng/ml for relapse.

A strength of our study is the virtually complete capture of RPs in the NPCR, which includes 98% of men with PCa in the Cancer Register, for which reporting is mandated by law [11]. We had access to longitudinal PSA measurements obtained after RP on a national level that provided us with a postoperative PSA trajectory for each man after RP [8]. To our knowledge, this is the first study to implement and assess a MLE missing data approach that handles the explicit censoring of PSA values below the detection limit when calculating PSA-DT.

This study has some limitations. It was restricted to men with a PSA relapse after RP, a clinical context characterized by low PSA levels and frequent values below the detection limit, so our findings may not be generalizable to other patients in whom PSA-DT could be performed differently. Performance of PSA-DT may depend on other factors, including how frequently PSA level is measured; however, assessing its impact was beyond the scope of this study. Our findings reflect clinical practice rather than a clinical trial where follow-up will be guided by a protocol. We did not perform multivariable modeling to assess the independent prognostic value of PSA-DT beyond other established predictors, and our analyses are therefore limited to unadjusted comparisons of prognostic performances. However, this approach reflects how PSA-DT is often considered in clinical practice, where it is typically interpreted alongside absolute PSA values rather than within formal multivariable models. Our cohort predominantly included men with low- and intermediate-risk disease, but the subgroup analysis of men with high-risk PCa indicated a similar pattern in prognostic performance. The use of contemporary imaging modalities, such as prostate-specific membrane antigen positron emission tomography at relapse and new treatment strategies after relapse, may affect the association between PSA-DT and PCa death, particularly in men with high-risk PCa.

Different methods to calculate PSA-DT may lead to different estimates of PSA-DT in various clinical settings [6], [7], [12]. We confirmed that this also applies to the setting of PSA relapse after RP, and found a wide individual-level range in estimates across estimation methods that differed in the approach to values below the detection limit and in the number of PSAs included. Though PSA-DT, as in previous studies [1], [2], [4], was discriminatory of PCa death, its discriminative ability in our study was comparable to PSA level at relapse. We are not aware of any previous systematic comparison of the ability of different PSA-DT estimators to discriminate PCa death or of a comparison of bias of the methods in simulations.

Our finding of within-individual variation of PSA-DT estimates shows that prognostic assessments based on the same PSA sequence will differ according to the method used. Some methods often produced negative PSA-DTs, for example, when using the two most recent PSAs with values of 0.22 and 0.21 ng/ml. Although we expected a priori that men with negative PSA-DT would have a favorable prognosis (since it indicates that PSA level is decreasing), men in our study with negative PSA-DTs only had a somewhat more favorable prognosis than men with a PSA-DT of 6–12 mo.

Previous studies have shown that PCa grows exponentially on a cellular level and that PSA grows exponentially in untreated men [13], [14]. It is, however, not evident that this applies after RP for PCa. Our findings of bias and wide range in PSA-DTs across different methods suggest that estimated PSA-DT does not necessarily represent a true progression rate, particularly when relapse is defined using a PSA cutoff of 0.1 ng/ml.

PSA-DT is used for risk stratification, according to the EAU guidelines, together with Gleason scores [2], [3], [4], [5]. However, our findings indicate that the absolute PSA level at relapse is equally discriminative and that the combination of this PSA with the Gleason score is a better prognostic marker, since it avoids the methodological difficulties of calculating PSA-DT [1], [4], [15], [16].

## 5 Conclusions

There was a wide within-individual range of PSA-DT across different estimation methods, and the absolute PSA level at relapse had a similar ability to discriminate PCa death as the estimates of PSA-DT. Given the methodological challenges associated with estimating PSA-DT, a prognostic approach based on PSA level at relapse, in combination with Gleason score, may be preferable in clinical practice.

  ***Author contributions***: Marcus Westerberg had full access to all the data in the study and takes responsibility for the integrity of the data and the accuracy of the data analysis.

  *Study concept and design*: Scilipoti, Callenmark, Westerberg.

*Acquisition of data*: Garmo, Stattin, Gedeborg, Westerberg.

*Analysis and interpretation of data*: Callenmark, Westerberg.

*Drafting of the manuscript*: Stattin, Westerberg.

*Critical revision of the manuscript for important intellectual content*: Scilipoti, Callenmark, Garmo, Stattin, Gedeborg, Westerberg.

*Statistical analysis*: Callenmark, Westerberg.

*Obtaining funding*: Stattin.

*Administrative, technical, or material support*: None.

*Supervision*: Westerberg.

*Other* (specify): None.

  ***Financial disclosures:*** Marcus Westerberg certifies that all conflicts of interest, including specific financial interests and relationships and affiliations relevant to the subject matter or materials discussed in the manuscript (eg, employment/affiliation, grants or funding, consultancies, honoraria, stock ownership or options, expert testimony, royalties, or patents filed, received, or pending), are the following:

  ***Funding/Support and role of the sponsor*:** This project was supported by The Swedish Research Council (2022-00544), The Swedish Cancer Society (22 2051), FORTE (2024-01652), and Uppsala University Hospital. The sponsors had no involvement with the planning, execution, or completion of the study.

  ***Acknowledgments*:** This project was made possible by the continuous work of the National Prostate Cancer Register of Sweden (NPCR) steering group: Elin Axén, Johan Styrke, Andreas Josefsson, Camilla Thellenberg, Hampus Nugin, Ingrida Verbiené, Stefan Carlsson, Anna Kristiansen, Mats Andén, Kimia Kohestani, Jon Kindblom, Thomas Jiborn, Olof Ståhl, Olof Akre, Eva Johansson, Magnus Törnblom, Fredrik Jäderling, Marie Hjälm-Eriksson, Lotta Renström Koskela, Erik Thimansson, Johan Stranne, Elin Trägårdh, Viktoria Gaspar, Fredrik Sandin, Petrus Stenson, Lena Pettersson, Mia Brus, Gustaf Hedström, Anna Hedström, Maria Moutran, Nina Hageman, and Maria Nyberg, and patient representatives, Hans Joelsson and Gert Malmberg.

  ***Ethics approval*:** PCBase Xtend has been approved by the Swedish Research Ethics authority as well as by the National Board of Health and Welfare and Statistics Sweden.

  ***Disclaimer:*** Rolf Gedeborg is employed by the Medical Products Agency (MPA) in Sweden. The MPA is a Swedish Government Agency. The views expressed in this article may not represent the views of the MPA.
