# Supplementary material for: Estimation and Prognostic Role of Prostate-specific Antigen (PSA) Doubling Time After Radical Prostatectomy
Source: Eur Urol Open Sci. 2026 May 21;89:1–9. doi: 10.1016/j.euros.2026.05.006 (PMC13218111; doi:10.1016/j.euros.2026.05.006)

Supplementary materials to

Estimation and prognostic role of prostate-specific antigen (PSA) doubling time after radical prostatectomy

Table of contents

Four definitions of relapse 2
Estimation of PSA doubling time 2
Simulation studies 3
Supplementary Table 1. 4
Supplementary Table 2. 5
Supplementary Figure 1. 7
Supplementary Figure 2. 8
Supplementary Figure 3. 9
Supplementary Figure 4. 10

Four definitions of relapse

We considered the following definitions of relapse

1. one value above 0.1 ng/mL,
2. one value above 0.2 ng/mL,
3. Two consecutive values above 0.1 ng/mL (main analysis), and
4. two consecutive values above 0.2 ng/mL.

All definitions are exemplified in **Supplementary Figure 1**. Relapse was only possible after the man had been PSA responsive defined as at least one PSA value ≤0.1 ng/mL within 42 days after surgery or the first PSA after 42 days ≤0.1 ng/mL. In all cases, men who did not initially have an undetectable PSA initiated non-adjuvant ADT or salvage radiotherapy before documented PSA relapse were not considered to have a PSA relapse.

Estimation of PSA doubling time

PSA doubling time was estimated using four different methods that all were based on a subset of all PSA values after the first PSA ≤0.1 ng/mL after surgery until and including the date of PSA relapse. All methods require at least two PSA values and were based on a linear regression model of the logarithm of the PSA values (logPSA) on time since surgery (months; 30 days)

logPSA = **Alpha** + **Beta** x **Time + Noise**

with coefficients **Alpha** and **Beta** and normally distributed and independent **Noise** with common standard deviation **Sigma**. This model was applied individually on each man.

- **Method 1 - Detectable PSAs**: all detectable PSAs above 0.1 ng/mL were used in the estimation. When relapse was based on one value above 0.1 ng/mL or 0.2 ng/mL there is no guarantee that there are at least two PSA values available for estimation so this method was only considered when relapse was based on two consecutive values above 0.1 ng/mL or 0.2 ng/mL.
- **Method 2 - Two most recent PSAs**: the two most recent PSAs were used. When relapse was defined based on one value above 0.1 ng/mL or 0.2 ng/mL the second last value (≤0.1 ng/mL) manually set to 0.1 ng/mL despite that the true value was unknown.
- **Method 3 - Three most recent PSAs**: the three most recent PSAs were used. When relapse was based on two values above 0.1 ng/mL or 0.2 ng/mL the third last PSA (≤ 0.1 ng/mL) was set to 0.1 ng/mL despite that the true value was unknown. When relapse was based on one value above 0.1 ng/mL or 0.2 ng/mL there is no guarantee that there are two detectable PSA values and one undetectable PSA value available for estimation so this method was only considered when relapse was based on two consecutive values above 0.1 ng/mL or 0.2 ng/mL.
- **Method 4 - All PSAs with censored maximum likelihood estimation (MLE)**: all PSA values were used, and undetectable PSA values (≤0.1 ng/mL) were handled as censored data (i.e. as ≤0.1 ng/mL) in a maximum likelihood estimation approach to the missing data.

The three first methods were all computed using regular least squares estimation using the R function *lm*. The method based on all PSAs was based on MLE where unknown PSA values (≤0.1 ng/mL) were considered as censored (a form of missing data). The censoring mechanism was assumed to be independent of the PSA values. The likelihood contribution of the censored data is the cumulative distribution function of the normal distribution evaluated at the cutoff 0.1 ng/mL with parameters equal to the estimated mean and variance. The R function *optim* was used to iteratively find the maximum likelihood estimates of the coefficients based on the *BFGS* algorithm and a finite difference approximation of the gradient.

Simulation studies

We performed a series of simulations where fictive PSA trajectories were simulated for 100 000 men according to an exponential growth curve model. The natural logarithm PSA value was simulated according to a normal distribution with a mean growing linearly with time: intercept + time x slope (doubling time = log(2)/slope). PSA at time 0 was set to a value below the detection limit 0.1 ng/mL, and PSAs were then sampled independently at 3 and 6 months and then the time between tests was distributed according to a normal distribution with mean 180 days and standard deviation 100 days but truncated to lie between 14 and 730 days.

We evaluated the bias of each estimator for three different intercepts (0.05, 0.025 and 0.005 ng/mL), four doubling times (6, 12, 18 and 24 months) and three standard deviations (0.05, 0.1, 0.15 ng/mL) and according to each of the four definitions of relapse.

Estimated PSA-DTs above 100 and below 0 months were set to 100 months since we expected that negative and extremely large doubling times would be interpreted similarly in clinical practice.

Supplementary Table 1. Follow-up and cause of death.

	One value above 0.1 ng/mL		Two consecutive values above 0.1 ng/mL (main analysis)		One value above 0.2 ng/mL		Two consecutive values above 0.2 ng/mL	
	N	(%)	N	(%)	N	(%)	N	(%)
**N men**	7691	(100)	6767	(100)	5468	(100)	3826	(100)
**Follow-up*** (years)								
Median (IQR)	6.2 (3.9 – 9.3)		6.0 (3.8 – 8.8)		6.0 (3.7 – 9.2)		5.7 (3.5 – 8.6)	
**Cause of death**								
Prostate cancer	204	(3)	185	(3)	178	(3)	145	(4)
Other causes	589	(8)	523	(8)	454	(8)	324	(8)
Alive at 10 years	6898	(90)	6059	(90)	4836	(88)	3357	(88)

*Estimated using the reverse Kaplan-Meier approach. IQR=inter-quartile range.

Supplementary Table 2. Baseline characteristics according to definition of relapse.

	One value above 0.1 ng/mL		One value above 0.2 ng/mL		Two consecutive values above 0.1 ng/mL		Two consecutive values above 0.2 ng/mL	
	N	(%)	N	(%)	N	(%)	N	(%)
**N men**	7691	(100)	5468	(100)	6767	(100)	3826	(100)
**Age at RP**								
2016-2024	66 (62-70)		67 (62-70)		66 (62-70)		67 (62-70)	
**Pathological Gleason**								
6-7	6463	(84)	4535	(83)	5676	(84)	3171	(83)
8-10	1228	(16)	933	(17)	1091	(16)	655	(17)
**PSA before RP** (ng/mL)								
Median (IQR)	7.7 (5.4-11.8)		7.7 (5.4-12)		7.7 (5.4-12)		7.8 (5.4-12)	
**Time to relapse** (months)								
Median (IQR)	27 (14-51)		34 (17-60)		31 (16 -54)		38 (20-66)	
**Number of tests between first undetectable PSA and relapse**								
Median (IQR)	6 (3-9)		7 (4-11)		7 (5-10)		9 (6-13)	
**Number of tests ≤ 0.1 ng/mL**								
Median (IQR)	5 (2-8)		4 (2-7)		5 (3-8)		4 (2-7)	
**PSA at relapse** (ng/mL)								
Median (IQR)	0.1 (0.1-0.2)		0.2 (0.2-0.3)		0.2 (0.1-0.2)		0.3 (0.2-0.4)	
**PSA doubling time** (months) – Detectable PSAs								
Median (IQR)	-		-		5.0 (0.2-12.9)		8.6 (3.8-16.7)	
<0	-	-	-	-	1688	(25)	252	(7)
0-12	-	-	-	-	3262	(48)	2178	(57)
>12	-	-	-	-	1817	(27)	1396	(36)
**PSA doubling time** (months) – Two most recent PSAs								
Median (IQR)	12.7 (6.2-29.7)		6.0 (3.4-9.9)		4.7 (-1.1-11.7)		4.4 (-0.8-11.8)	
<0	0	(0)	0	(0)	1749	(26)	980	(26)
0-12	3671	(48)	4496	(82)	3359	(50)	1907	(50)
>12	4020	(52)	972	(18)	1659	(25)	939	(25)
**PSA doubling time** (months) – Three most recent PSAs								
Median (IQR)	-		-		11.8 (6.4-22.8)		7.1 (4.1-11.7)	
<0	-	-	-	-	3	(0)	3	(0)
0-12	-	-	-	-	3418	(51)	2908	(76)
>12	-	-	-	-	3346	(49)	915	(24)
**PSA doubling time** (months) – All PSAs with censored MLE								
Median (IQR)	10.9 (5-26.3)		6.6 (3.4-12.5)		8.1 (4.1-16.6)		7.9 (4-14.1)	
<0	0	(0)	0	(0)	1	(0)	0	(0)
0-12	4092	(53)	4027	(74)	4386	(65)	2630	(69)
>12	3599	(47)	1441	(26)	2380	(35)	1196	(31)

	One value above 0.1 ng/mL		Two consecutive values above 0.1 ng/mL		One value above 0.2 ng/mL		Two consecutive values above 0.2 ng/mL	
	N	(%)	N	(%)	N	(%)	N	(%)
**N men**	7691	(100)	6767	(100)	5468	(100)	3826	(100)
**Age at RP**								
2016-2024	66 (62-70)		66 (62-70)		67 (62-70)		67 (62-70)	
**Pathological Gleason**								
6-7	6463	(84)	5676	(84)	4535	(83)	3171	(83)
8-10	1228	(16)	1091	(16)	933	(17)	655	(17)
**PSA before RP** (ng/mL)								
Median (IQR)	7.7 (5.4-11.8)		7.7 (5.4-12)		7.7 (5.4-12)		7.8 (5.4-12)	
**Time to relapse** (months)								
Median (IQR)	27 (14-51)		31 (16 -54)		34 (17-60)		38 (20-66)	
**Number of tests between first undetectable and relapse**								
Median (IQR)	6 (3-9)		7 (5-10)		7 (4-11)		9 (6-13)	
**Number of tests ≤ 0.1 ng/mL**								
Median (IQR)	5 (2-8)		5 (3-8)		4 (2-7)		4 (2-7)	
**PSA at relapse** (ng/mL)								
Median (IQR)	0.1 (0.1-0.2)		0.2 (0.1-0.2)		0.2 (0.2-0.3)		0.3 (0.2-0.4)	
**PSA doubling time** (months) – Detectable PSAs								
Median (IQR)	-		5.0 (0.2-12.9)		-		8.6 (3.8-16.7)	
<0	-	-	1688	(25)	-	-	252	(7)
0-12	-	-	3262	(48)	-	-	2178	(57)
>12	-	-	1817	(27)	-	-	1396	(36)
**PSA doubling time** (months) – Two most recent PSAs								
Median (IQR)	12.7 (6.2-29.7)		4.7 (-1.1-11.7)		6.0 (3.4-9.9)		4.4 (-0.8-11.8)	
<0	0	(0)	1749	(26)	0	(0)	980	(26)
0-12	3671	(48)	3359	(50)	4496	(82)	1907	(50)
>12	4020	(52)	1659	(25)	972	(18)	939	(25)
**PSA doubling time** (months) – Three most recent PSAs								
Median (IQR)	-		11.8 (6.4-22.8)		-		7.1 (4.1-11.7)	
<0	-	-	3	(0)	-	-	3	(0)
0-12	-	-	3418	(51)	-	-	2908	(76)
>12	-	-	3346	(49)	-	-	915	(24)
**PSA doubling time** (months) – All PSAs with censored MLE								
Median (IQR)	10.9 (5-26.3)		8.1 (4.1-16.6)		6.6 (3.4-12.5)		7.9 (4-14.1)	
<0	0	(0)	1	(0)	0	(0)	0	(0)
0-12	4092	(53)	4386	(65)	4027	(74)	2630	(69)
>12	3599	(47)	2380	(35)	1441	(26)	1196	(31)

Supplementary Figure 1. Illustration of definitions of PSA relapse.

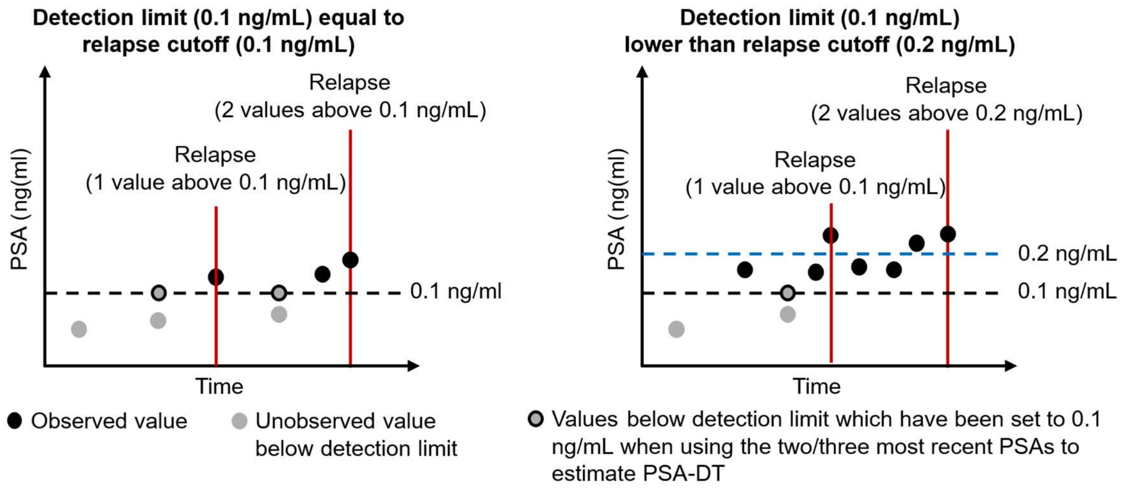

Supplementary Figure 2. Study flow chart.

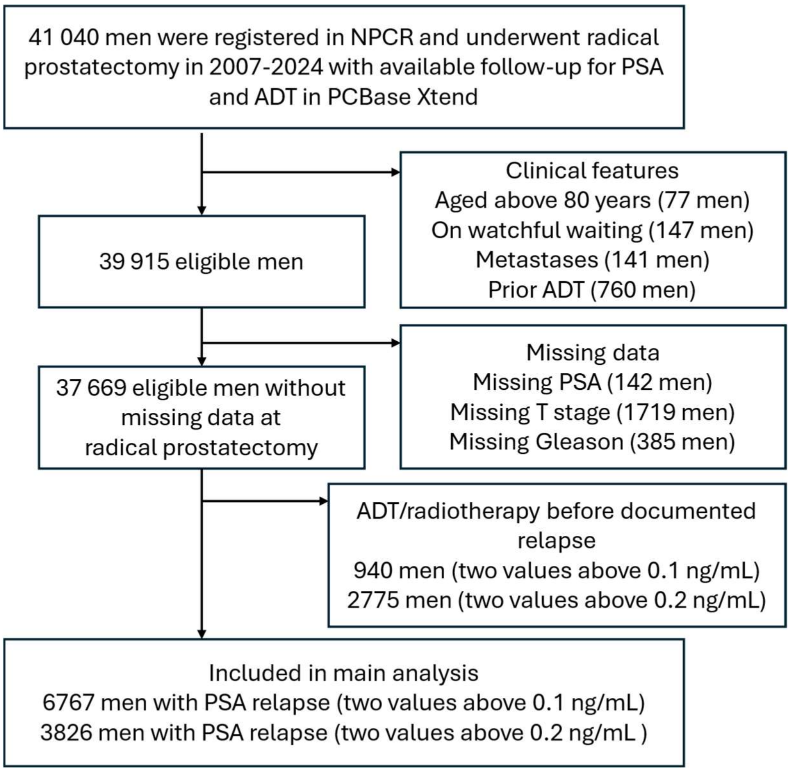

NPCR=National Prostate Cancer Register of Sweden

PCBase Xtend=Prostate Cancer database Sweden with with Extended Treatment and Endpoint Data

PSA=Prostate-specific antigen

ADT=Androgen deprivation therapy

Supplementary Figure 3. Discrimination of prostate cancer mortality within 10 years from relapse using alternative definitions of relapse based on one value. Grey indicates that negative PSA doubling times were set to the maximum (100 months) and black indicates that negative PSA doubling times remained negative. Men were censored if they died from other causes.

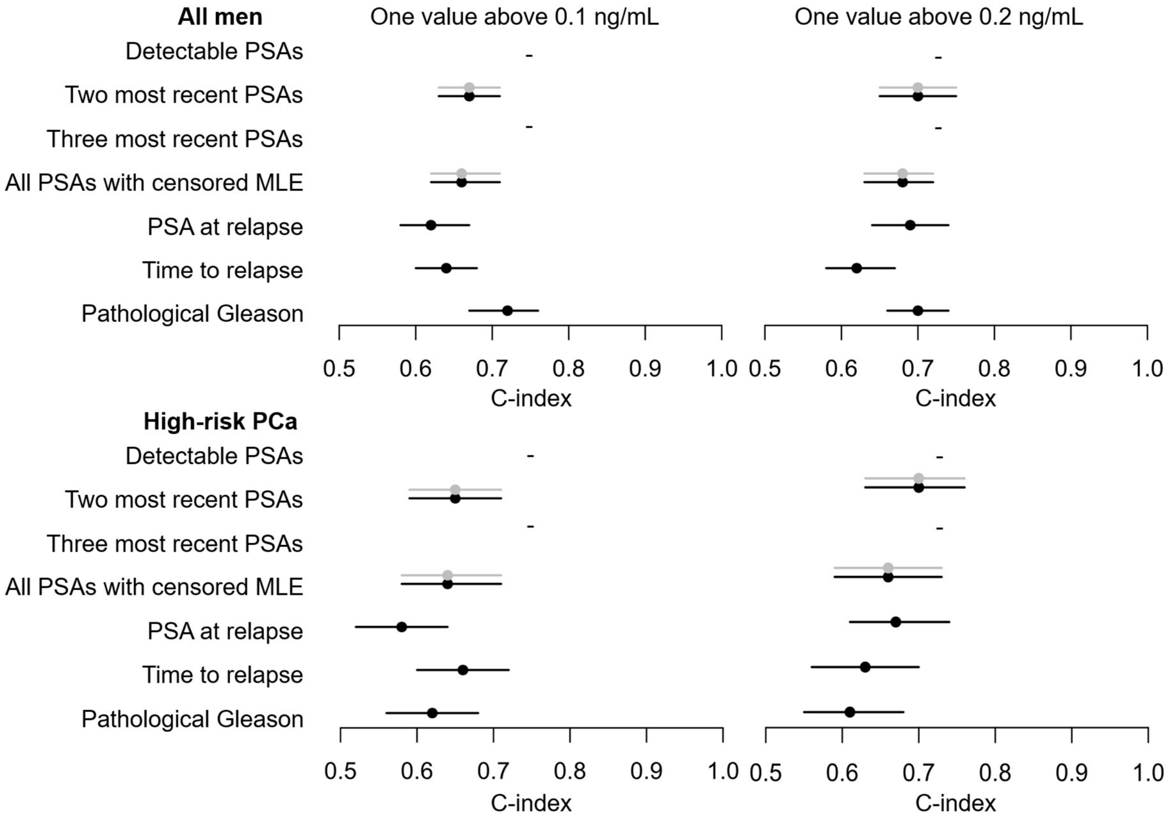

Supplementary Figure 4. Bias of each estimator in all simulation studies according to PSA doubling time. When relapse was defined based on one value above 0.1 ng/mL or 0.2 ng/mL the methods based on detectable PSAs and the three most PSAs was not able to estimate PSA doubling time for some simulated individuals (because only one value above the detection limit was available). PSA doubling time was truncated at 100 months and negative doubling times were set to 100 months.

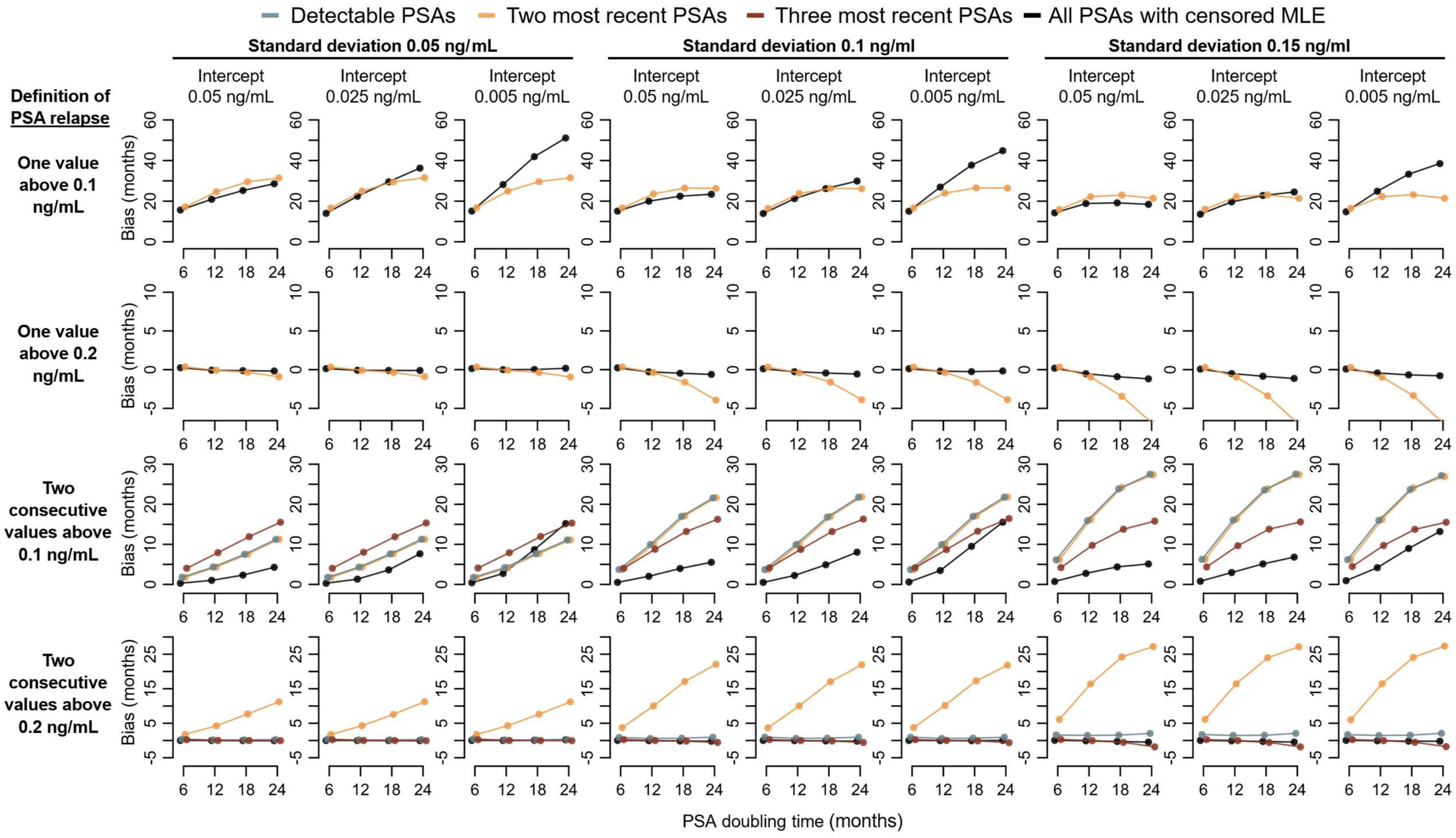

Supplement: Supplementary Data 1 [file mmc1.pdf]
